# Proteomics study on the protective mechanism of soybean isoflavone against inflammation injury of bovine mammary epithelial cells induced by *Streptococcus agalactiae*

**DOI:** 10.1007/s12192-020-01158-1

**Published:** 2020-08-31

**Authors:** Hui Niu, Hua Zhang, Fuxin Wu, Benhai Xiong, Jinjin Tong, Linshu Jiang

**Affiliations:** 1 Department of Animal Science, Animal Science and Technology College, Beijing University of Agriculture, Beijing, 102206 China; 2 State Key Laboratory of Animal Nutrition, Institute of Animal Science, Chinese Academy of Agricultural Sciences, Beijing, 100193 China

**Keywords:** Soybean isoflavone, Dairy mammary epithelial cells, *Streptococcus agalactiae*, Protection mechanism, Proteomics

## Abstract

This study aimed to verify the anti-inflammatory effect of soybean isoflavones (SI) on the inflammatory response induced by *Streptococcus agalactiae* (*S. agalactiae*) of bovine mammary epithelial cells (bMECs) and to elucidate its possible mechanism. BMECs were pretreated with SI of different concentrations (20, 40, 60, 80, 100 μg/mL) for 0.5, 3, 6, 9, 12, 15, 18, 24 h. And then, *S. agalactiae* was used to infect bMECs for 6 h (MOI = 50:1) to establish the inflammation model. Cell viability, growth curves of *S. agalactiae*, cytotoxicity, and *S. agalactiae* invasion rate were determined. A proteomics technique was used to further detect differential proteins and enrichment pathways. SI (40 μg/mL) improved the viability of bMECs at 12 h (*p* < 0.05) and 60 and 80 μg/mL of SI greater (*p* < 0.01). Moreover, 60 μg/mL of SI protects cells from bacterial damage (*p* < 0.05). SI could inhibit *S. agalactiae* growth and internalization into bMECs in a time- and dose-dependent manner. In addition, proteomics results showed that 133 proteins were up-regulated and 89 proteins were down-regulated significantly. The differentially significantly expressed proteins (DSEPs) were mainly related to cell proliferation, differentiation, apoptosis, and migration. GO annotation showed that 222 DSEPs were divided into 23 biological processes (BP) terms, 14 cell components (CC) terms, and 12 molecular functions (MF) terms. DSEPs were significantly enriched in 10 pathways, of which the immune pathway was the main enrichment pathway.

## Introduction

*S. agalactiae* is one of the common pathogens causing subclinical mastitis in dairy cows (Santos et al. 2011). Mammary epithelial cells, as the first line of defense against mastitis, play an important role in the process of resisting the invasion of pathogenic microorganisms (Bougarn et al. 2011). After dairy cows are infected with *S. agalactiae*, bacteria in the infected mammary gland gradually increase. In the case of continuous infection, somatic cell count in milk is increasing, and mammary gland tissue is further damaged. The acini in the gland finally lose their complete structure, and the blood milk barrier is destroyed, causing inflammation (Patil et al. 2015). Abundant research indicated that *S. agalactiae* could induce significantly increased expression of inflammatory factors, such as TNF-α, IL-6, and IL-8, which promotes the progress of inflammation (Adams Waldorf et al. 2011).

Soybean isoflavones are kinds of flavonoids existing in legume plants (Tsugami et al. 2017). In recent years, some studies suggest that SI has played important roles in the treatment of human diseases, such as anti-tumor, anti-inflammatory, and anti-oxidative stress (Mortensen et al. 2009; Russo et al. 2016; Teixeira et al. 2014). Moreover, the insulin-like growth factor (IGF-I) content in the blood of dairy cows increased by adding SI into the diet. JAK-STAT and mTOR signal pathways are influenced by IGF-I, which regulate the expression of milk protein-related genes to promote milk protein synthesis and increase milk yield (Sigl et al. 2014). However, the effect of SI on the inflammatory response induced by *S. agalactiae* of bMECs has not been reported.

Proteomics technology has become an effective approach to reveal the occurrence and prevention mechanism of many diseases (Vaccaro et al. 1993). Tandem mass tags (TMT) can be covalently combined with free amino acids of lysine and the N-termini of peptides to tag a variety of polypeptides in the sample, so as to make quantitative analysis more accurate. High sensitivity proteomics technology based on TMT can significantly improve the experimental flux, reduce the quantitative error, and find more subtle biological differences. In addition, it is appropriate for differential protein analysis of samples with multiple processing methods or from multiple processing times.

The objectives of the present study were to evaluate the potential of SI to protect bMECs from *S. agalactiae* infection and reveal the underlying mechanism at the proteomics level. We determined the effect of SI on bMECs activity, *S. agalactiae* growth, and bacteria invasion into cells, as well as analyzed the function of DSEPs in the protection of SI with TMT-coupled LC-MS/MS, and potential biochemical markers were identified from the experimental data, which provided a new direction to prevent the inflammation of bMECs induced by *S. agalactiae*.

## Material and methods

### Cell culture and treatment

In this experiment, bovine mammary epithelial cells (Gifts from the Laboratory of Animal Biochemistry and Molecular Biology, Northeast Agricultural University) were used. The growth medium was composed of DMEM/F12 medium (Gibco, USA), 10% Australian fetal bovine serum (FBS, Gibco, USA), and 1% penicillin-streptomycin (Gibco, USA). BMECs were cultured in the medium and maintained in a humidified incubator at 37 °C in an atmosphere of 5% CO_2_. The medium was replaced with fresh medium every 24 or 48 h until the cells were thoroughly distributed across the bottom of the dish. A confluent monolayer of the cell culture was treated with a 0.25% trypsin-EDTA solution (Gibco, USA) for 3–5 min and neutralized by adding DMEM/F12 supplemented with 10% FBS. Single cells were collected from the suspension after centrifugation using an Allegra™ 6R centrifuge (Sigma, USA) at 4*g*, 25 °C, for 5 min.

For subsequent tests, BMECs were plated on 96-well plates (Corning, USA) at a density of 1 × 10^3^ cells/well and cultured in the growth medium until 80–90% confluency. The cells were washed twice with D’Hanks buffer without antibiotics and the growth medium was replaced with the assay medium (DMEM/F12 supplemented with 10% FBS without antibiotics) containing different concentrations of SI. Cells were pretreated with various concentrations of SI (0, 20, 40, 60, or 80 μg/mL) and maintained in the humidified incubator for various incubation periods (at 0.5, 3, 6, 9, 16, 12, 15, 18, and 24 h).

### Bacterial strains and growth conditions

*S. agalactiae* (CVCC3940) was purchased from Veterinary Medicine Supervision Institute of China and inoculated in the brain heart infusion (BHI, Gibco, USA) medium at 37 °C for 24 h.

### Verification of the model of SI against bMEC inflammation induced by *S. agalactiae*

### Cell viability assay

The viability of bMECs was determined using the CCK-8 assay according to the manufacturer’s instructions (Beyotime Biotechnology, Shanghai, China). The cells were distributed into 96-well plates and mixed with SI according to the experimental design. After the specific incubation period, 20 μL of CCK-8 reagent was added to each well and incubated at 37°C for 2°h. Cell viability in each well was measured by the optical density (OD) at 450°nm using a microplate reader (Bio Tek, USA). The experiments were performed in triplicate for each sample.

### Growth curves

To evaluate the kinetics of the antimicrobial effects, the growth curves of *S. agalactiae* were drawn. Bacteria were plated on 96-well plates containing BHI medium at 1 × 10^5^ CFU/well. SI was added to each well at concentrations of 0, 40, 60, and 80 μg/mL, respectively, and the OD value at 600 nm was determined at 0, 3, 6, 9, 12, and 24 h. The experiments were performed in triplicate for each sample.

### Cytotoxicity assay

The cytotoxicity of *S. agalactiae* on bMECs with or without SI was quantified using a lactate dehydrogenase (LDH) assay kit (Beyotime Biotechnology, Shanghai, China). The cell treatment was performed based on previous tests. Briefly, the cells were treated with SI at various concentrations (0, 40, 60, and 80 μg/mL) for 12 h. Subsequently, *S. agalactiae* was added to cells at the multiplicity of infection (MOI) = 50:1, followed by incubation at 37 °C, 5% CO_2_ for 6 h, as reported previously (Mingwei et al. 2020a, 2020b). The content of LDH was detected with a microplate reader at 490 nm. The experiments were performed in triplicate for each sample.

### *S. agalactiae* invasion assay

Bacterial suspensions were prepared with DMEM/F12 at an MOI = 50:1. The bMECs grown in 24-well plates were infected with bacterial suspensions and treated with different concentrations of SI (0, 40, 60, or 80 μg/mL) at 37 °C, 5% CO_2_ for 2 h. BMECs were washed 5 times with sterile PBS, and 250 μg/mL gentamicin was added, followed by incubation at 37 °C, 5% CO_2_ for 0.5 h. BMECs were washed 5 times again with sterile PBS to remove the gentamicin. Next, cells were lysed with 0.5% Triton-X-100 (Solarbio, Beijing, China) for 15 min. The number of CFU was determined by the standard colony counting technique on BHI solid medium. The experiments were performed in triplicate for each sample.

### Protein extraction and digestion

The cells were washed three times by 3 mL of precooled PBS and treated with 800 μL of RIPA lysis buffer containing 1/10 protease inhibitor cocktail, then were placed on ice. After 10 min, the cells were collected into a 1.5-mL centrifuge tube and placed in liquid nitrogen. The protein concentration was determined by BCA Protein Assay Kit (Beyotime Biotechnology, Shanghai, China) according to the manufacturer’s instruction. In order to identify whether the protein supported the requirements of the follow-up experiment, 20 μg protein was taken from each sample for SDS-PAGE analysis as described by Wiśniewski et al. (2009)).

Aliquots of 100 μg of protein in each sample were mixed with 800-μL RIPA lysis buffer and 10 mM tris-(2-carboxyethyl) phosphine (TCEP) and incubated at 37 °C for 1 h. The protein mixture was incubated at 37 °C in the dark for 40 min after treatment with 40 mM iodoacetamide. Each protein sample was mixed with 600 μL of precooled acetone and incubated at − 20 °C for 4 h. The protein was obtained by centrifugation at 10,000*g* for 20 min, and then was mixed with 100 mM triethyl ammonium bicarbonate (TEAB) after removing supernatant. Next, trypsin was added at 1:50 trypsin-to-protein mass ratio and incubated at 37 °C overnight.

One unit of TMT reagent was thawed and reconstituted in 50 μL acetonitrile. After tagging for 2 h at room temperature, hydroxylamine was added and allowed to react for 15 min at room temperature. After that, the peptide segments were added to 50 μL ultra pure water and incubated at room temperature for 30 min. The same number of labeled products in each group was mixed in a tube and dried in a vacuum concentrator. The polypeptide samples were redissolved in the buffer solution of UPLC and separated in high pH liquid phase by reversed phase C18 column. Finally, liquid-phase tandem mass spectrometry was used to detect differential proteins.

### Protein database searching and analysis

The raw files produced from LC-MS/MS were imported into MaxQuant software (http://www.maxquant.org) (version 1.6.1.0). Then, the transcriptome database was used for data interpretation and protein identification. The title of reference was transcriptome data of *Epinephelus fuscoguttatus* infected by Vibrio vulnificus (Jazamuddin et al. 2018). The protein database was sourced from http://www.ncbi.nlm.nih.gov/sra/SRX3067303. MaxQuant search parameters were set as follows: isobaric labels, TMT 10 plex; reporter mass tolerance, 0.005 Da; max missed cleavages, 2; first search peptide tolerance and MS/MS tolerance, 20 ppm; fixed modifications, carbamidomethyl (C); variable modifications, oxidation (M), acetyl (Protein N-term); and false discovery rate (FDR), < 0.01. Razor and unique peptides were used for protein quantification.

The mass spectrometry proteomics data have been deposited in the ProteomeXchange Consortium (http://proteomecentral.proteomexchange.org) via the iProX partner repository (Ma et al. 2019) with the dataset identifier PXD018816.

### Bioinformatics analysis

Perseus software and R statistical computing software were used to analyze the bioinformatics data (Zhang et al. 2019). DSEPs were screened with the cutoff of a ratio fold-change of > 1.20 or < 0.83 and *p* values < 0.05. Combining the comparative analysis of variance (ANOVA), *t* test, and FDR (Benjamin–Hochberg), all qualitative and quantitative protein analysis results were obtained. Hierarchical clustering was adopted to categorize expression data together according to the protein level. Huge amounts of data are produced by mass spectrometry technology in proteomics, which represents all the biological processes of the organism. The aim of bioinformatics analysis was to find the source and mechanism for biological changes. Gene Ontology (GO) enrichment, Kyoto Encyclopedia of Genes and Genomes (KEGG), and protein interaction network analysis were adopted (Tyanova et al. 2016).

### Statistical analysis

Results were reported as mean ± SEM. The data were analyzed with a one-way ANOVA using IBM SPSS Statistics 21 software (IBM, Armonk, NY). Differences with *p* < 0.05 were considered statistically significant, and *p* < 0.01, extremely significant.

## Results

### Protection of SI on bMECs against inflammation induced by *S. agalactiae*

#### Improving the viability of bMECs

**Fig. 1:**
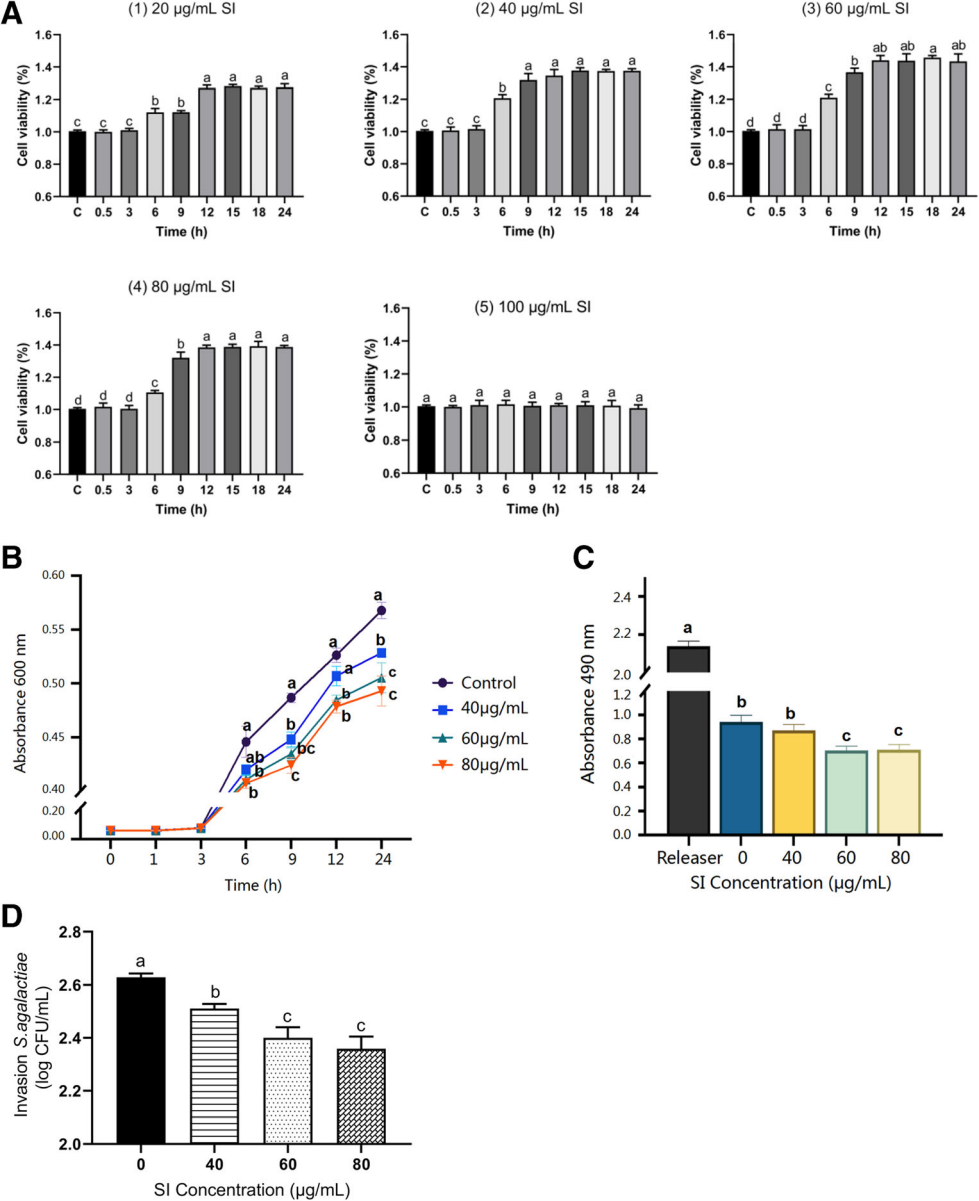
Effects of soybean isoflavone on bMECs infected with *S. agalactiae.* **A** Viability of bMECs exposed to (1) 20, (2) 40, (3) 60, (4) 80, and (5) 100 μg/mL of soybean isoflavone (*n* = 4). Letters (a–d) represent a significant difference within each sample group (*p* < 0.05), and bars represent SEM. **B** Growth curves of *S. agalactiae* exposed to different concentrations of soybean isoflavone. Means at the same time (h) point with different letters (a–c) differ significantly for treatment effect (*n* = 4). **C** Cytotoxicity of *S. agalactiae* on bMECs after exposure to soybean isoflavone (*n* = 4). Letters (a–c) represent a significant difference within each sample group (*p* < 0.05). **D** The number of intracellular *S. agalactiae* after exposure to soybean isoflavone (*n* = 3). Data are presented as the means ± SEM. Letters (a–c) represent a significant difference within each sample group (*p* < 0.05)

Figure 1 A shows that the viability of bMECs was improved with certain concentrations of SI in a time-dependent manner. Compared with the control group, cell viability was hardly changed at 0.5 h and 3 h. However, cell viability was significantly improved at 12–24 h with 20 μg/mL of SI (*p* < 0.05). And cell viability was significantly improved at 6 or 9 h in the 40-, 60-, or 80-μg/mL SI-treated groups (*p* < 0.05). Subsequently, an obvious (*p* < 0.05) increase in cell viability occurred, and the effect of 60 or 80 μg/mL of SI on cell viability enhancement was even more significant (*p* < 0.01). However, there was no significant difference in cell viability at 12, 15, 18, and 24 h. Therefore, SI concentrations of 40, 60, and 80 μg/mL and interaction time of 12 h were chosen to explore the protective effects of SI against bMEC inflammation induced by *S. agalactiae*.

#### Inhibiting the growth of *S. agalactiae*

As shown in Fig. 1B, there was an inhibitory effect on *S. agalactiae* growth with SI of different concentrations (40, 60, 80 μg/mL). We observed that SI started to show antibacterial effect at 3 h compared with the control group. Bacterial growth was significantly (*p* < 0.05) inhibited by 60 and 80 μg/mL of SI, and the inhibition of *S. agalactiae* by 80 μg/mL of SI was stronger. In general, the apoptosis of bMECs induced by *S. agalactiae* was reduced by SI in a time- and dose-dependent manner.

#### Reducing bMEC damage induced by *S. agalactiae*

According to our previous findings, the experimental model of bMECs infected with *S. agalactiae* was established after treatment with SI for 12 h (Fig. 1C). In comparison with the control group, 60 μg/mL of SI significantly (*p* < 0.05) down-regulated the content of LDH, which minimized cell injury induced by *S. agalactiae*. This injury decreased significantly (*p* < 0.05) when the concentration of SI was 80 μg/mL.

#### Inhibiting *S. agalactiae* internalization into bMECs

As shown in Fig. 1D, 40, 60, and 80 μg/mL of SI inhibited *S. agalactiae* internalization to cells to different extents. The results showed that different concentrations of SI could inhibit *S. agalactiae* internalization into bMECs, 40 μg/mL of which significantly (*p* < 0.05) reduced the invasion, and 60, 80 μg/mL of which significantly (*p* < 0.01) reduced the invasion.

### Proteomic analysis

#### Differentially significantly expressed proteins

**Fig. 2:**
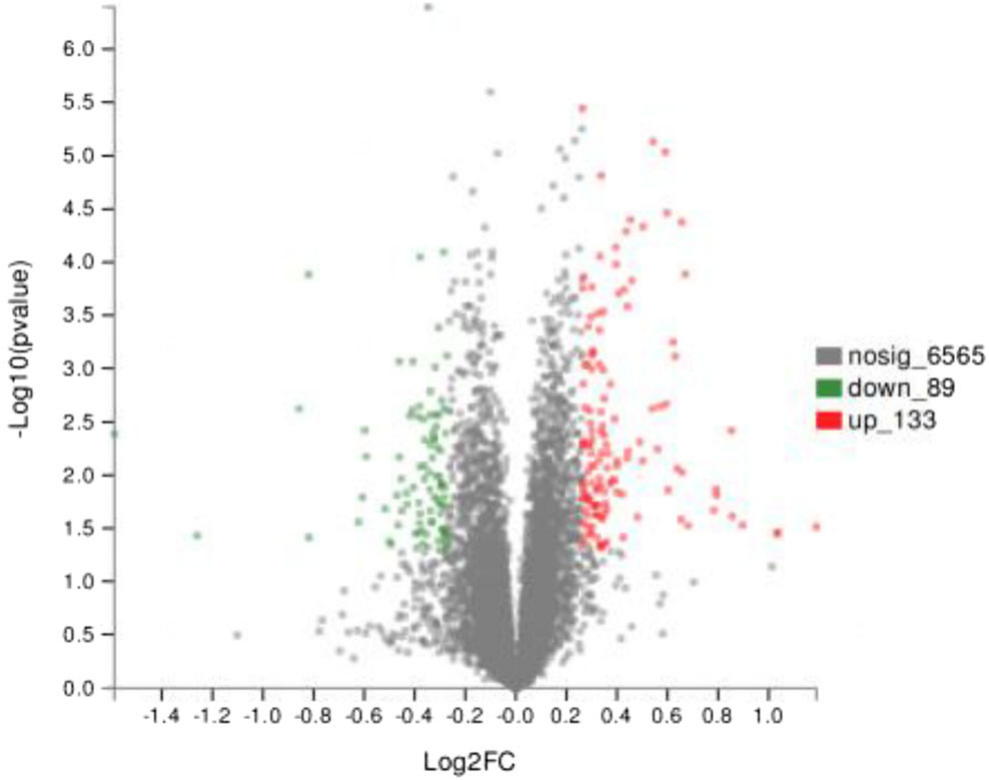
Volcano plots of DSEPs between soybean isoflavone group and the control group. The abscissa is Log2 fold change, indicating the multiple change value of protein expression difference between the two groups. The vertical coordinate is *P* value, indicating the significance of different changes in gene expression. Red dots indicate significantly up-regulated proteins, green dots indicate significantly down-regulated proteins, and gray dots indicate non-significant differentially abundant protein

The volcano plots showed the changes of protein expression with and without SI protecting bMECs from *S. agalactiae* infection. Through statistical analysis with R software, 6787 proteins were screened out, and 222 had significant changes (*p* < 0.05). A total of 133 up-regulated DSEPs (fold change > 1.2) were identified and are shown as red dots in Fig. 2. Eighty-nine down-regulated DSEPs (fold change < 0.83) were identified and represented as green dots in Fig. 2.

Some notable DSEPs are shown in Tables 1 and 2. Among the up-regulated differential proteins, the expression of some enzymes increased significantly, including cyclin-dependent kinase inhibitor 1 (p27kip1), lipid phosphate phosphohydrolase 1 (LPP-1), hydroxyacyl-coenzyme A dehydrogenase, mitochondrial (SCHAD), acetyl-coenzyme A synthetase, cytoplasmic (ACSS2), phosphatidate phosphatase LPIN1 (PAP-LPIN1), and glutamine synthetase (GS). Among the down-regulated proteins, integrin beta-5 (ITG-β5), osteopontin (OPN), phospholipase D2 (PLD2), eukaryotic translation initiation factor 1b (elFs), and GTP-binding nuclear protein Ran (Ran/TC4) were changed dramatically.

**Table 1:** Information about some significantly up-regulated proteins in the SI group compared with control group

Protein	Description	NCBI Accession	FC (SI/CON)	Log2FC (SI/CON)	*P* value (SI/CON)
Cyt C	Cytochrome c	XP_010802623.1	1.228	0.296	< 0.001
p27kip1	Cyclin dependent kinase inhibitor 1	XP_005223383.1	1.513	0.598	< 0.001
PIK3CA	PIP3	DAA33278.1	1.211	0.277	0.015
CD82	CD82 antigen isoform X1	XP_015330346.1	1.253	0.326	0.019
LPP-1	Lipid phosphate phosphohydrolase 1	DAA17913.1	1.231	0.300	0.001
SCD1	Stearoyl-CoAdesaturase	NP_776384.3	1.486	0.571	0.002
SCHAD	Hydroxyacyl-coenzyme A dehydrogenase, mitochondrial	DAA28877.1	1.216	0.283	0.046
FABP3	Fatty acid-binding protein3	NP_776738.1	1.208	0.273	0.021
ACSS2	Acetyl-coenzyme A synthetase, cytoplasmic	XP_005214643.1	1.233	0.302	0.005
jun D	Jun D proto-oncogene	DAA28179.1	1.258	0.331	0.001
CLU	Clusterin	XP_024851197.1	1.204	0.268	0.001
PAP-LPIN1	Phosphatidate phosphatase LPIN1	XP_015329639.1	1.416	0.502	0.007
GS	Glutamine synthetase	NP_001035564.1	1.316	0.397	< 0.001
HES-1	Transcription factor HES-1	NP_001029850.1	1.810	0.826	0.025

**Table 2:** Information about some significantly down-regulated proteins in the SI group compared with control group

Protein	Description	NCBI accession	FC (SI/CON)	Log2FC (SI/CON)	*P* value (SI/CON)
SKP1	Sphingosine kinase 1	DAA18164.1	0.775	− 0.368	0.044
ITGβ5	Integrin beta-5	XP_024847121.1	0.820	− 0.286	< 0.001
OPN	Osteopontin	XP_024848751.1	0.812	− 0.301	0.010
PLD2	Phospholipase D2	NP_001069295.1	0.815	− 0.295	0.032
EPCR	Endothelial protein C receptor	XP_024856089.1	0.762	− 0.392	0.049
A1P	Alpha-1-antiproteinase precursor	NP_776307.1	0.657	− 0.606	0.016
Ran / TC4	GTP-binding nuclear protein Ran	NP_001029877.1	0.823	− 0.280	0.037
elFs	Eukaryotic translation initiation factor 1b	XP_024838403.1	0.806	− 0.312	0.006
PRAS40	PRAS proline-rich 40	XP_005219317.1	0.769	− 0.379	< 0.001
PF4/CXCL4	Platelet factor 4	DAA28540.1	0.333	− 1.588	0.004

### Go function classification and enrichment analysis

**Fig. 3:**
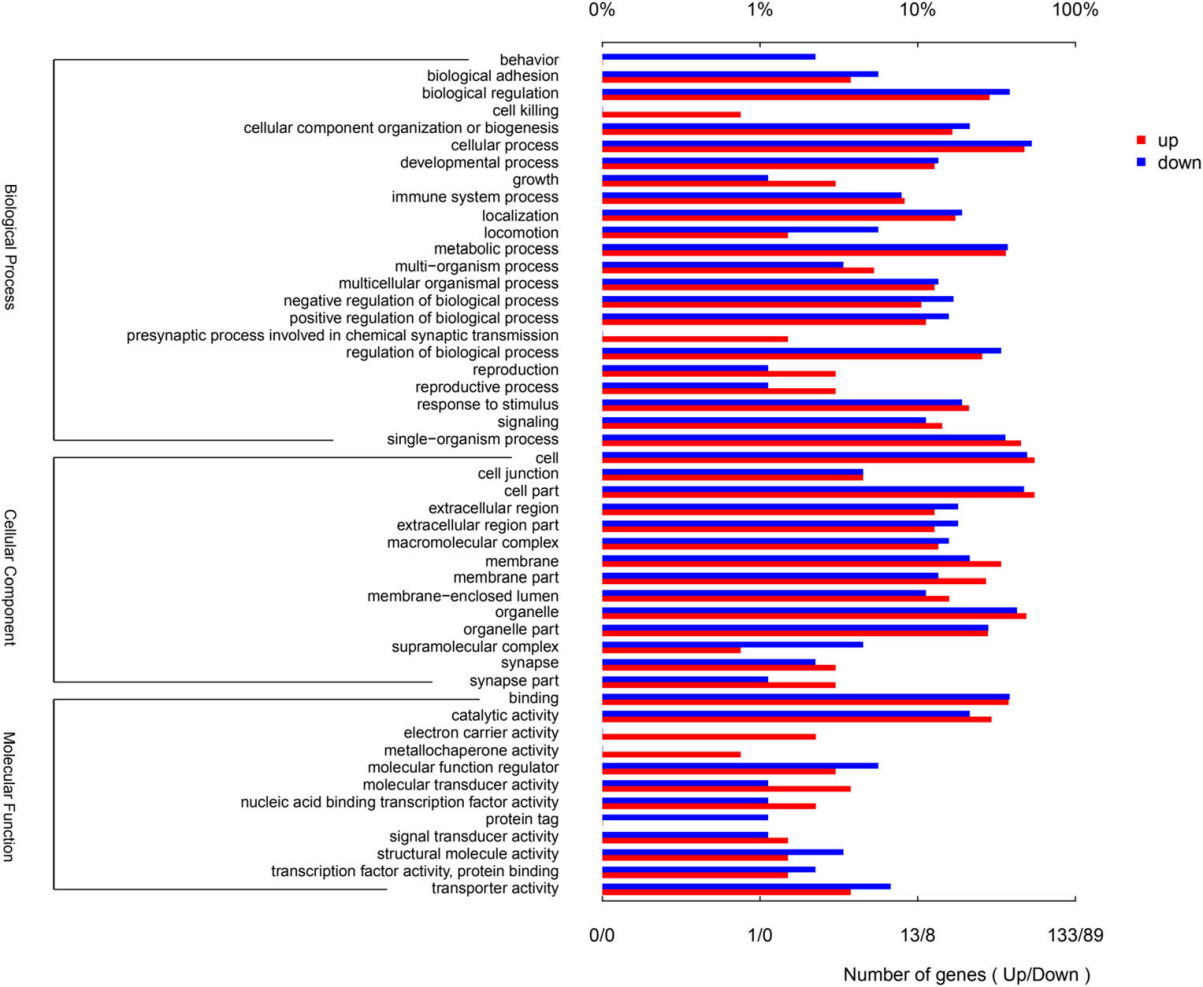
GO terms of the DSEPs of soybean isoflavone group compared with control group. The red bars represented the up-regulated DSEPs, and blue bars represented down-regulated DSEPs

The differential proteins were classified by Gene Ontology (http://www.geneontology.org/) according to their biological processes, cell components, and molecular functions. Gene ontology results showed that 222 DSEPs were classified into 49 GO terms, including 23 BP terms, 14 CC terms, and 12 MF terms (Fig. 3). Compared with the control group, the DSEPs were involved in almost all biological processes. However, up-regulated DSEPs were not involved in behavior, and down-regulated DSEPs were not involved in cell killing and presynaptic process involved in chemical synaptic transmission. The main BP included biological regulation, cellular process, metabolic process, regulation of biological process, and single-organism process. In the CC ontology, the DSEPs were mainly concentrated in cell, cell part, membrane, membrane part, organelle, and organelle part. In addition, the DSEPs were mainly related to the binding, catalytic activity, and other molecular functions. Similarly, the up-regulated DSEPs were not related to protein tag, while down-regulated DSEPs were not related to electron carrier activity and metallochaperone activity.

### KEGG pathway analysis

**Fig. 4:**
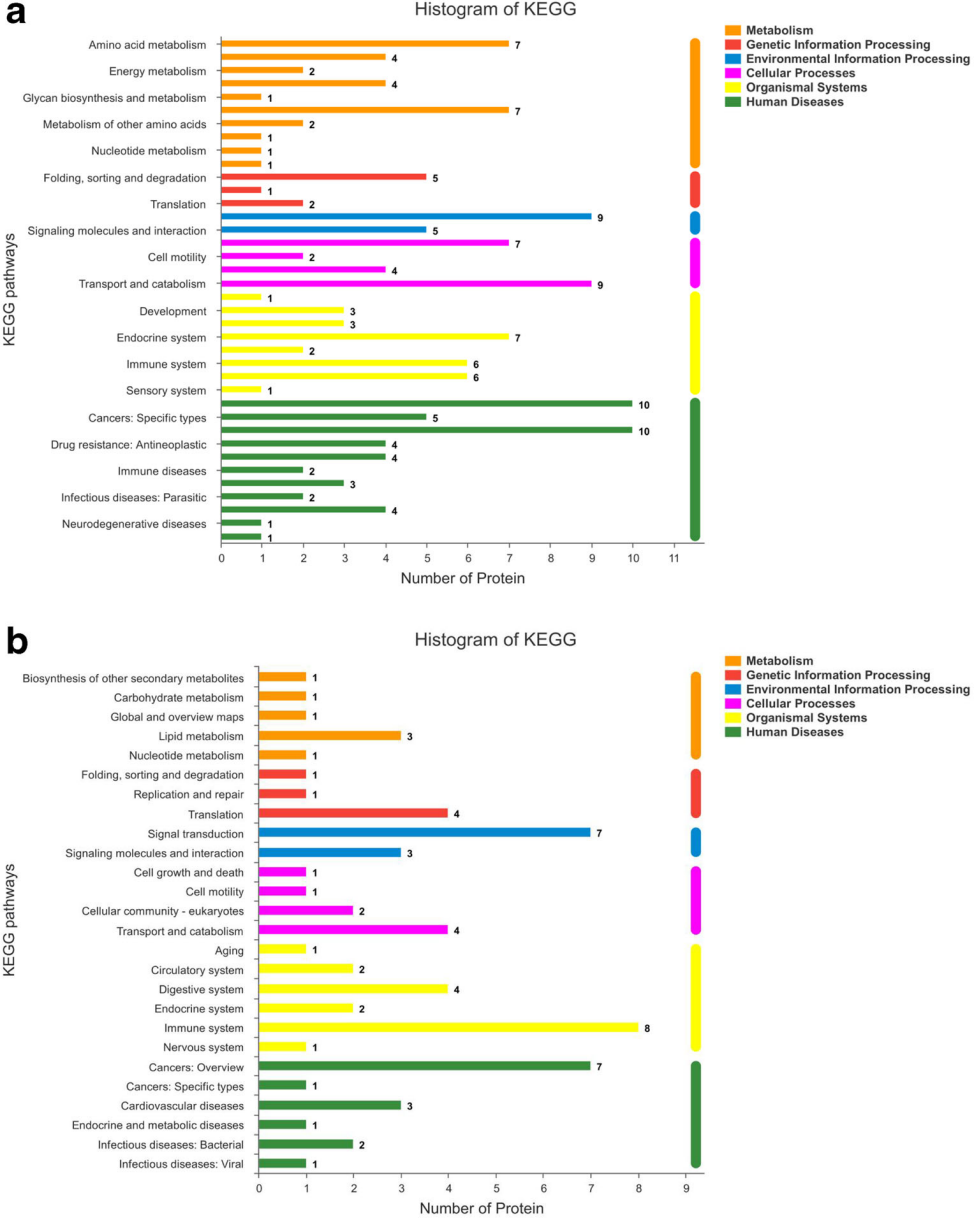
KEGG pathway analysis of DSEPs. The ordinate is the name of KEGG pathway, and the abscissa is the number of proteins annotated to this pathway. **a** KEGG annotation analysis of up-regulated DSEPs. **b** KEGG annotation analysis of down-regulated DSEPs

The pathway annotation analysis of DSEPs is helpful to confirm the function of proteins. The annotation results of DSEPs were classified according to the type of KEGG pathway, as shown in Fig. 4a, b. The up-regulated DSEPs are enriched in 38 pathways, including 10 metabolism pathways, 3 genetic information processing pathways, 2 environmental information processing pathways, 4 cellular process pathways, 8 organismal system pathways, and 11 human disease pathways. The pathways most closely affected by soybean isoflavones are amino acid metabolism, lipid metabolism, signal transduction, cell growth and death, transport and catabolism, endocrine system, cancers: overview, cardiovascular disease. The down-regulated DSEPs were mainly enriched in 26 pathways, among which signal transduction, immune system pathway, and cancers: overview pathway was the main enrichment pathways. These significantly enriched pathways played important roles in protecting bMECs from *S. agalactiae*. A single protein may be involved in multiple pathways during KEGG annotation.

**Fig. 5:**
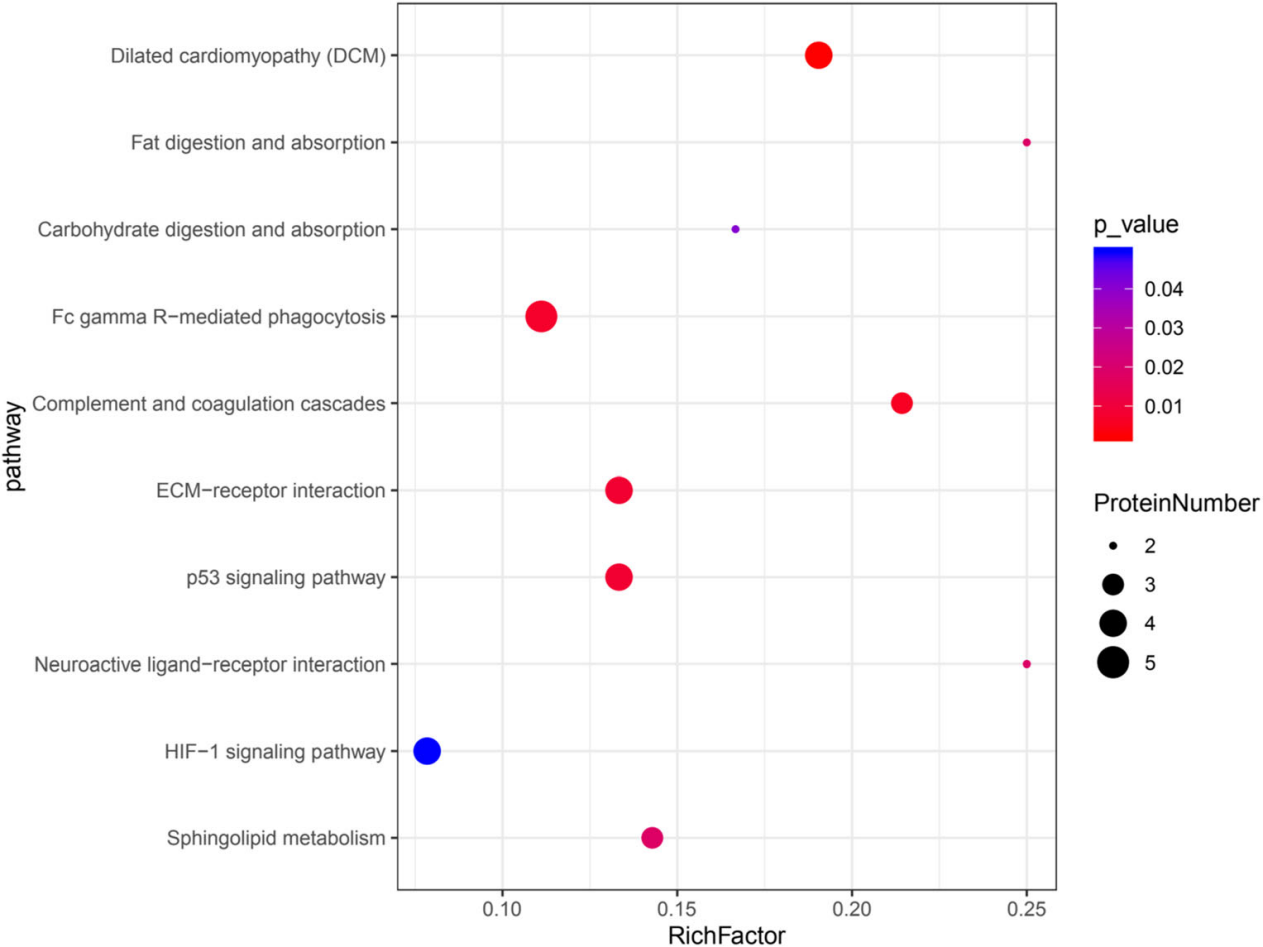
The Kyoto Encyclopedia of Genes and Genomes (KEGG) enrichment pathways of DSEPs

The KEGG pathway analyses revealed that the DSEPs were significantly enriched in 10 pathways after removing the enrichment pathway related to human diseases (Fig. 5). Among them, two pathways are related to immunity, such as Fc gamma R-mediated phagocytosis and complement and coagulation cascades. Actually, dilated cardiomyopathy (DCM) is also related to the immune pathway, because the change of immune response can change the susceptibility of the host to the disease, which leads to myocardial autoimmune injury. There are two pathways related to digestion, including fat digestion and absorption as well as carbohydrate digestion and absorption, two pathways related to metabolism, including HIF-1 signaling pathway and sphingolipid metabolism, and two pathways related to environmental information processing and signaling molecules and interaction, such as neuroactive ligand–receptor interaction and ECM–receptor interaction. The P53 signaling pathway is one of the main signaling pathways of apoptosis.

## Discussion

It has been reported that the anti-inflammatory activity and antimicrobial properties of SI can affect dairy cows (Fiordalisi et al. 2016; Gohlke et al. 2013; Mozaffarian and Wu 2018) and alter the rumen or gut microbiota (Amaretti et al. 2015; Espley et al. 2014; Zhan et al. 2017). Furthermore, *S. agalactiae* as the most important contagious pathogens was the most frequently isolated pathogen in cows with subclinical mastitis, as it was detected in 2.8% of 3288 clinical mastitis samples in Chinese dairy cows (Gao et al. 2017). Intramammary infection (IMI) with *S. agalactiae* triggers a complex host immune response that involves immune cells. Especially, the mammary gland epithelial cells were demonstrated to be capable of responding to bacterial invasion and initiating an inflammatory immune reaction (Pu et al. 2017). Therefore, understanding the mechanisms of the soybean isoflavone protection effect response to *S. agalactiae* infection is important for the development of innovative strategies for mastitis prevention or treatment.

In this study, we proved that soybean isoflavone has an anti-inflammatory effect on the inflammation of bMECs induced by *S. agalactiae*, in line with previous reports about their antimicrobial and antioxidant properties (Ravishankar et al. 2013). Moreover, the activity of mammary epithelial cells was significantly increased with different concentrations of SI at 12 h (Fig. 1) in a time-dependent manner. Previous studies have shown that contagious pathogens appear to give rise to different host immune-related gene and protein signatures. *Streptococcus agalactiae* and *Staphylococcus aureus* are major causes of mastitis and have been well-studied (Jensen et al. 2013; Vidanarachchi et al. 2015). However, there is little information pertaining to *S. agalactiae*-induced inflammation on mammary gland epithelial cells, in particular, the protein change response to the effect of soybean isoflavone addition.

TMT-labeled proteomics was used to analyze the different proteins of bMECs infected by *S. agalactiae* in the presence or absence of soybean isoflavone. Under the action of SI, p27kip1 protein (also known as CDK1 protein inhibitor) was significantly up-regulated. P27kip1 is a CDK inhibitor of cyclin, which plays an important role in the regulation of cell proliferation and cell cycle (Polyak et al. 1994). It was reported that the up-regulation of p27kip1 can prevent the G1 to S phase cell cycle transition, thus inhibiting cell proliferation. The down-regulation of p27kip1 protein can cause abnormal cell proliferation, which is closely related to carcinogenesis (Polyak et al. 1994). Therefore, we proved that soybean isoflavone may regulate the cell cycle by up-regulating p27kip1 protein to avoid abnormal cell proliferation. PIK3CA protein, also known as PIP3 (phosphatidylinositol-3, 4, 5-triphosphate), is the second messenger on the plasma membrane, which acts mainly through the PI3K-Akt-mTOR signal pathway (Bhattachara et al. 2013). Studies have shown that the up regulation of PIK3CA protein can promote the growth and transformation of mammary epithelial cells and inhibit apoptosis (Wen et al. 2014). As a transmembrane protein, CD82 plays an important role in cell invasion, especially in cell movement. The expression of CD82 can inhibit the secretion of IL-8, and the binding of CD82 transmembrane protein and chemokine receptor can interrupt the signal transduction of IL-8 in endothelial cells (Khanna et al. 2014). Interleukin-8 (IL-8), a proinflammatory cytokine, can activate inflammatory cells and promote the process of inflammatory response (Rasmussen et al. 2008). It is valid to consider that SI can significantly up-regulate the expression of CD82 protein, thus inhibiting the secretion of IL-8 and weakening the occurrence of the cellular inflammatory response. Therefore, all these up-regulated proteins play an important role in the bMEC defense against the *S. agalactiae* infection and response to the effect of soybean isoflavone.

Integrins not only mediate cell-to-cell adherence and immune cell migration, but also take part in signal transduction (Lee et al. 2000). The expression of integrin 5 (ITG-β5) was decreased significantly in the present study. Integrin, as a cell adhesion protein, plays an important role in regulating adhesion, proliferation, and movement in cell and extracellular environment (Pasqualini and Hemler 1994). It is the communicator between cells and their extracellular environment and can activate growth receptor and downstream cell signals. Some studies suggest that *Streptococcus agalactiae* can bind to mammary epithelial cells through ITG-β5 and stimulate ITG-β5 as its receptor to enter cells, participate in, and aggregate to trigger a series of signaling pathways of epithelial cells (Zheng et al. 2016). It was reported that the expression of ITGA5 is also increased in many solid primary mammary tumors and it promotes tumor cell growth and survival (Miroshnikova et al. 2017). Thus, the finding that ITGA5 was down-regulated implied a possible relationship of ITGA5 in mastitis inhibition by SI treatment in dairy cows.

Osteopontin (OPN), which can stimulate the expression of SRC and focal adhesion kinase (FAK) and promote their phosphorylation, was also decreased in response to the soybean isoflavone treatment. Both SRC and FAK are non-receptor protein tyrosine kinases, which can be indirectly located at the site of integrin receptor through C-terminal domain-mediated integrin-related protein, activate SRC after integrin combines with OPN, activate SRC after activation phosphorylates FAK, and thus promote cell migration (Li et al. 2007). Therefore, SI may inhibit the expression of FAK by inhibiting the secretion of ITG-β5 and OPN, so as to reduce the ability of *S. agalactiae* to invade the bovine mammary epithelial cells and achieve the protective effect of soybean isoflavone on the mastitis induced by *Streptococcus agalactiae*.

KEGG enrichment analysis showed that the difference protein was significantly enriched in the immune pathway. Immune function can resist or prevent microbial infection and keep cells in a healthy state. For example, Fc gamma R-mediated phagocytosis is related to immune function, which mediates actin binding and phagocytosis formation. The pathogenic microorganisms that invade the body can be killed and digested in the phagocyte (Yamada et al. 1989). In addition, the complement and coagulation cascades are critical components of the innate immune defense against pathogens, which is a primary line of defense against infection (Yang et al. 2017; Zhang et al. 2018). Thus, the KEGG results support our hypothesis that SI is important in host defense against pathogens and other pathogen pathways and is the main defense line against infection. The ECM–receptor interaction and focal adhesion pathway events culminate in the reorganization of the actin cytoskeleton, which is a prerequisite for cell transformation and migration (Morita et al. 2007). In particular, ECM–receptor interaction is important in maintaining the morphogenesis of tissues and organs and the structure of cells and tissues (Huang et al. 2014). It can be speculated that SI can promote the adhesion between cells and ECM, resist the stimulation of foreign pathogens, and protect against inflammatory damage through regulating the ECM–receptor interaction. Therefore, the significant changes in proteins related to these pathways may be an important part of the mechanism whereby SI reduces tissue damage caused by *S. agalactiae*-induced inflammation.

In conclusion, soybean isoflavone (40, 60, 80 μg/mL) promoted bMEC viability after treatment with 60 μg/mL for 12 h, which could minimize cytotoxicity. SI inhibited *S. agalactiae* growth and internalization into bMECs in a dose-dependent manner. The targets of SI against inflammation injury of bMECs induced by *S. agalactiae* might be proteins related to cell proliferation, migration, and adhesion, such as P27kip1, PIK3CA, CD82, ITG-β5, and OPN. SI mainly regulates cell immune function to resist microbial infection so as to keep the cells in a healthy state. Therefore, highlighting candidate protein response to SI treatment may be useful for diagnosis and defense against mastitis.
